# Genome-Wide Identification, Characterization and Expression Analyses of Heat Shock Protein-Related Genes in a Highly Invasive Ascidian *Ciona savignyi*

**DOI:** 10.3389/fphys.2018.01043

**Published:** 2018-07-31

**Authors:** Xuena Huang, Shiguo Li, Yangchun Gao, Aibin Zhan

**Affiliations:** ^1^Research Center for Eco-Environmental Sciences, Chinese Academy of Sciences, Beijing, China; ^2^University of Chinese Academy of Sciences, Chinese Academy of Sciences, Beijing, China

**Keywords:** adaptation, biological invasion, *Ciona*, environmental stress, gene expression, heat shock protein

## Abstract

Biological response to rapid changing environments is an outstanding research question in ecology and evolution. Biological invasions provide excellent “natural” experiments to study such a complex response process, as invaders often encounter rapidly changing environments during biological invasions. The regulation of heat shock proteins (Hsp) is a common pathway responsible for various environmental stresses; however, the comprehensive study on Hsp system across the whole genome and potential roles in determining invasion success are still largely unexplored. Here, we used a marine invasive model ascidian, *Ciona savignyi*, to investigate transcriptional response of Hsp-related genes to harsh environments. We identified 32 genes, including three Hsp20, six Hsp40, ten Hsp60, eight Hsp70, three Hsp90, one Hsp100, and one heat shock transcription factor (Hsf), across the whole genome of *C. savignyi*. We further characterized gene structure and protein motifs, and identified potential heat shock elements (HSEs) in promoters of Hsp genes. The expression analysis showed that most Hsp genes, but not all, were involved in transcriptional response to temperature and salinity challenges in a duration- and stress-specific pattern, and the maximum amplitude of induction occurred in Hsp70-4 after 1-h of high temperature treatment. However, the Hsf gene was scarcely induced and limited interactions were predicted between Hsp and Hsf genes. Our study provide the first systematic genome-wide analysis of Hsp and Hsf family in the marine invasive model ascidian, and our results are expected to dissect Hsp-based molecular mechanisms responsible for extreme environmental adaptation using *Ciona* as a model system.

## Introduction

Nowadays, species are frequently subjected to various suboptimal environmental challenges owing to multiple factors such as global climate change, range expansion (e.g., biological invasions), and environmental pollution caused by human activities (Hoffmann and Sgrò, 2011; Zhan et al., 2015). The ability of species’ rapid adaptation to changing environments is crucial for their maximal survival, particularly for sessile organisms who cannot actively escape from environmental stresses (Skelly et al., 2007; Hoffmann and Sgrò, 2011). Adaptive responses can occur through natural selection upon genetic variation originated from standing variations or new mutations (i.e., evolutionary adaptation), and epigenetic modifications without changing underlying genetic basis (i.e., plastical adaptation; Artemov et al., 2017). Thus, deep investigations on rapid adaptation to environmental challenges, particularly to extreme environments that species frequently encounter now, are critical to assess impacts of rapid environmental changes on species’ geographical distributions and evolutionary fates (Seneviratne et al., 2014).

Biological invasions provide excellent “natural” experiments to study molecular mechanisms responsible for environmental stresses, as invasive species usually encounter dramatically different environments in recipient habitats and/or rapidly changing environments during the process of invasions (Colautti and Lau, 2015; Zhan et al., 2015; Briski et al., 2018). Although great efforts have been made to investigate the response mechanisms to environmental stresses during biological invasions, such a scientific question has not been well answered, mainly owing to multiple interacting factors and/or pathways involved (Zhan et al., 2010, 2012; Bock et al., 2015; Huang et al., 2017). Among known underlying mechanisms for rapid and flexible stress responses, heat shock proteins (Hsp), as a group of evolutionarily conserved protein families, is one of the crucial molecular pathways that facilitate organisms to cope with environmental fluctuations through correcting protein folding and/or maintaining protein homeostasis (Feder and Hofmann, 1999; Morimoto, 2002). Hsp are generally classified into six major families based on molecular mass: small heat shock protein (sHsp) family with molecular mass from 10 to 30 kD (also known as Hsp20 family), as well as larger molecular mass families including Hsp40, Hsp60, Hsp70, Hsp90, and Hsp100/110 (Evgen’ev et al., 2014). Initially, the genes coding for Hsp70 were identified as chromosome puffs in *Drosophila* after heat shock treatment in 1962, and subsequently accumulating evidence on Hsp functions suggests that Hsp could be induced by not only heat stresses but also many other abiotic and/or biotic challenges, such as osmotic pressure, heavy metal, ultraviolet radiation, oxidative stress, and bacteria infection (Sørensen et al., 2003). Although the relationship between induced Hsp expression and stress resistance has been widely studied and confirmed in an array of species, the stress response processes associated with Hsp are still complicated and not well systematically resolved, mainly due to the following aspects: (1) not all Hsp members can be induced by stresses, and some constitutively expressed Hsp also play important roles for survival under non-stressful conditions (Borchel et al., 2017), (2) different Hsp members with distinct molecular structures showed diverse functions in response to the same types of stresses (Fujikawa et al., 2010), (3) the induction of Hsp expression by different stresses may vary with stress types and/or intensities (Cellura et al., 2006; Dong et al., 2008), (4) the roles of Hsp in adapting to the same stressful environments are species-specific (Serafini et al., 2011), (5) mechanisms of Hsp expression are multi-layered regulated, including transcriptional control by binding of heat shock factor (Hsf) to heat shock elements (HSEs) at the promoter region of heat shock genes, as well as at the translation and even DNA methylation levels (Serafini et al., 2011; Pu and Zhan, 2017), and (6) individuals living in distinct environments often use different strategies for adaptation, such as preadaptation and/or up-regulation of Hsp expression in response to stresses (Gleason and Burton, 2015). As a result, systematic identification of a complete set of Hsp and Hsf genes across the whole genome and comprehensive investigation of their response patterns to environmental stresses are necessary for clarifying the adaptive roles of Hsp in ecological and evolutionary studies. So far, this work has been well conducted in model species, such as *Mus musculus*, *Danio rerio*, *Caenorhabditis elegans*, and *Drosophila* (Borchel et al., 2017). However, it remains largely unexploited in aquatic invaders, where they encounter frequently extreme environments during invasions (Zhan et al., 2015). The comprehensive study of Hsp-related genes in invasive species, as well as their roles in invasion success, can help dissect ecological and evolutionary mechanisms responsible for extreme environmental adaptation.

*Ciona savignyi*, a notorious invasive marine invader with its native ranges in Japan and possibly northern Asia, has widely invaded the Pacific coast of North America, Atlantic coast of Argentina and even Southern Hemisphere such as New Zealand (Smith et al., 2010; Zhan et al., 2015). This ascidian species has become a model to study invasion success in aquatic ecosystems, mainly owing to multiple characteristics such as survival in a wide range of temperatures/salinities and small well-sequenced genome (160 MB) (Vinson et al., 2005; Zhan et al., 2015). During its range expansions, multiple environmental stresses, especially water temperature and salinity fluctuations, have been observed in *C. savignyi* (Zhan et al., 2015), and Hsp may get involved in response to stressful environments and facilitate this species to survive and subsequently invade new areas with varied environmental factors.

Here, we identified the complete set of Hsp and Hsf based on the sequenced genome of *C. savignyi*, characterized the gene structure and protein motifs, and compared all these features with its sister species *C. robusta*. Subsequently, we tested expression changes of all identified Hsp and Hsf under temperature and salinity stresses to understand their roles in response to stressful environments at the transcriptional level. Finally, in order to elucidate the regulatory mechanisms of stress response, we identified the potential HSE in the promoter region of each Hsp and further analyzed the correlation between Hsp and Hsf expression. Our results are expected to elucidate ecological and evolutionary roles of Hsp-related genes in response to temperature and salinity stresses during invasions by this model invasive ascidian, as well as to provide important clues to study the evolution of Hsp due to the unique evolutionary position of genus *Ciona* between invertebrates and vertebrates (Procaccini et al., 2011).

## Materials and Methods

### Sequences Retrieval and Phylogenetic Reconstruction

To compare the compositions of Hsf and Hsp families between *C. savignyi* and its congener *C. robusta*, all potential genes were identified in these two species in parallel. The proteomes of *C. savignyi* (Proteome ID: UP000008144) containing 20,004 protein sequences and *C. robusta* (Proteome ID: UP000007875) containing 17,309 protein sequences were downloaded from UniProt website^1^. Subsequently, we downloaded the Hidden Markov Model (HMM) profile files of Hsp20 (PF00011), Hsp40 (PF01556), Hsp60 (PF00118), Hsp70 (PF00012), Hsp90 (PF00183), and Hsf (PF00447) from the Pfam database^2^. Searches for six domains against *C. savignyi* and *C. robusta* proteome databases were conducted by HMMER 3.0 with an E-value < 1e^-6^ (Finn et al., 2011). The obtained candidate Hsp and Hsf proteins were further examined for the presence of conserved domains with Pfam (see footnote 2) and SMART databases^3^. The gene names of Hsp and Hsf consist of three aspects: species information (Cs = *Ciona savignyi*; Cr = *Ciona robusta*), and Hsp family and order number were sequentially assigned randomly. The full length amino acid sequences of Hsp60, Hsp70, and Hsp90 families in *Drosophila melanogaster*, *Caenorhabditis elegans*, *Branchiostoma floridae*, *Petromyzon marinus*, *Danio rerio*, and *Xenopus laevis* were aligned separately by ClustalW, and then the maximum likelihood (ML) phylogenetic trees were constructed, respectively, using MEGA 6.0 software with Jones-Taylor-Thornton (JTT) amino acid substitution model and 1,000 bootstrap replicates. *D. melanogaster* Hsp genes was used to visualize the phylogenetic structures and motif compositions, mainly because Hsp were initially found in this species and its Hsp have been comprehensively studied.

### Analysis of Subcellular Localization, Gene Structure, and Conserved Motifs

The theoretical molecular weight (Mw) and isoelectric point (pI) of putative Hsp and Hsf proteins were predicted by the pI/Mw tool^4^. Subcellular localizations were predicted using WoLF PSORT^5^. The gene structures of all candidate genes were illustrated using Gene Structure Display Server 2.0 (GSDS^6^) by aligning the coding sequences with the corresponding genomic DNA sequences from the same genes. The conserved motifs in proteins were analyzed by MEME online program^7^ with the following parameters: number of repetitions = any, maximum number of motifs = 20, and optimum motif width = 30–70 residues.

### *Cis*-Element Prediction and Protein–Protein Interaction Analysis

The transcription factor binding sites were predicted at 1,000 bp upstream and 1,000 bp downstream of transcription start site (TSS) of Hsp using MatInspector tool in Genomatix Software Suite^8^. Here, we only focused on potential HSE recognized by Hsf genes (5′-nnGAAnnTTCnnGAAnn-3′), and we further investigated the size, number and position of HSE following the criteria proposed by Tian et al. (2010) based on previously identified HSE. Protein–protein interaction (PPI) among Hsp and Hsf was detected by STRING^9^ with the confidence score > 0.8.

### Biological Materials and Stress Treatments

Adult *C. savignyi* were collected from the coast of Dalian, Liaoning Province, China (38°49’13”N, 121°24’20”E). The procedures of acclimation, daily feeding, and stress treatments were performed according to Huang et al. (2016). Briefly, field environments (temperature of 15°C and salinity of 30aaa) were used to set as the control group, and other four challenged groups included high temperature (HT: 25°C and salinity: 30aaa), low temperature (LT: 5°C and salinity: 30aaa), high salinity (HS: 40aaa and temperature: 15°C) and low salinity (LS: 20aaa and temperature: 15°C). Pharynx tissue was dissected out after 1-, 24-, and 48-h exposure for each treatment, and all collected samples were immediately frozen in liquid nitrogen, and then stored at -80°C until gene expression analysis.

### Gene Expression Analysis

Total RNA was extracted from all samples (three biological replicates for each sample) by the TRIZOL reagent, and transcriptome library construction and standard sequencing were achieved by Illumina sequencing with pair end of 150 bp strategy using the HiSeq4000 platform. The subsequent processes including mapping to reference genome, assembling and gene expression analysis were carried out by the pipeline of HISAT, StringTie, and Ballgown (Pertea et al., 2016). The differential expression analysis were conducted between each of the four treatment groups and control group at the same time point, and the fold change (FC) value refers to the multiple of the expression of a gene in treatment group relative to control group. The statistical analysis of *P*-value was implemented by the linear model test with Ballgown. The fragments per kilo-base per million reads (FPKMs) values and FC values of all Hsp and Hsf genes were extracted from the RNA-seq results for further analyses. Principal component analysis (PCA) was conducted to visualize the overall difference between treatment groups and control group at the same time points using FPKM values of all Hsp and Hsf genes. The heatmap for expression profiles were shown using log fold change values *via* pheatmap package under the R environment. Dynamic trends of temporal expression changes at three time points were examined by the short time-series expression miner (STEM) program, by which expression profiles would be significantly enriched by comparing the distribution of observed groups with those expected in a random permutation (Ernst and Bar-Joseph, 2006).

## Results

### Identification of Potential Hsp and Hsf Genes

We found and validated a total of 32 putative Hsp and Hsf genes after searching relevant HMM profiles against *C. savignyi*’s proteome. Due to a high level similarity between Hsp100 and Hsp70 families (Lee-Yoon et al., 1995), one Hsp70 gene obtained by using Hsp70 HMM profile was re-assigned to Hsp100 gene family based on the predicted molecule weight of 100 kD. The rest of 31 genes included three Hsp20, six Hsp40, ten Hsp60 (including a novel predicted gene Hsp60-9), eight Hsp70, three Hsp90, and one Hsf (**Table 1**). The prediction of subcellular localization revealed that the only CsHsf acting as transcriptional factor was predicted to be localized in the nucleus, while CsHsp were present in various cytosolic compartments, mostly in cytoplasm and some in endoplasmic reticulum (ER), mitochondria and nucleus (**Table 1**). Although a total of 10 CsHsp60 were identified, BLAST analysis revealed that nine cytosolic CsHsp60 showed high similarity to eight different subunits (α, β, γ, δ, 𝜀, ζ, η, and 𝜃) of chaperonins containing T-complex polypeptide 1/TCP1 ring complex (CCT/TRiC), which formed hetero-oligomers to function as a whole, while the remaining mitochondrial CsHsp60-8 represented another subfamily of chaperonins.

**Table 1:** Summary information of the Hsf and Hsp families in *C. savignyi.*

Gene name	Gene ID	AA/MW/PI	Genomic locus	Subcellular localization
Hsf	ENSCSAVG00000009385	478/53.799/5.10	reftig_90: 222,523–223,959	Nucl
Hsp20-1	ENSCSAVG00000000921	193/21.408/5.87	reftig_312: 2,232–5,579	Cyt_ Nucl
Hsp20-2	ENSCSAVG00000000281	217/25.248/6.25	reftig_401: 102,219–105,165	Nucl
Hsp20-3	ENSCSAVG00000005534	92/10.174/7.8	reftig_13: 1,062,933–1,063,499	Cyt_ Nucl
Hsp40-1	ENSCSAVG00000006976	350/39.785/8.71	reftig_133: 809,856–812,974	Nucl
Hsp40-2	ENSCSAVG00000007731	217/24.300/9.19	reftig_60: 1,120,943–1,122,012	Cyt_ Nucl
Hsp40-3	ENSCSAVG00000003847	403/45.319/6.48	reftig_35: 634,966–640,598	Cyt
Hsp40-4	ENSCSAVG00000008972	404/46.384/8.78	reftig_156: 374,311–380,085	Cyt
Hsp40-5	ENSCSAVG00000008657	325/35.659/9.10	reftig_26: 1,311,834–1,318,124	Cyt
Hsp40-6	ENSCSAVG00000009142	381/42.974/9.00	reftig_48: 906,751–907,896	Cyt
Hsp60-1	ENSCSAVG00000004234	546/60.739/6.71	reftig_21: 629,188–631,061	Cyt
Hsp60-2	ENSCSAVG00000005041	544/60.205/6.44	reftig_60: 147,642–149,276	Cyt
Hsp60-3	ENSCSAVG00000008004	543/59.472/6.37	reftig_26: 1,230,151–1,236,766	Cyt
Hsp60-4	ENSCSAVG00000000012	540/59.365/7.62	reftig_451: 111,719–120,981	Cyt
Hsp60-5	ENSCSAVG00000003875	539/58.952/5.86	reftig_13: 310,857–312,476	Cyt
Hsp60-6	ENSCSAVG00000009661	536/58.046/8.31	reftig_90: 354,806–357,327	Cyt
Hsp60-7	ENSCSAVG00000008115	576/62.425/5.50	reftig_77: 2,940,210–2,949,992	Cyt
Hsp60-8	ENSCSAVG00000000669	597/63.920/5.38	reftig_247: 178,990–187,341	Mito
Hsp60-9	Novel gene	559/60.097/6.78	reftig_12: 172,148–173,993	Cyt
Hsp60-10	ENSCSAVG00000000743	537/58.655/5.29	reftig_239: 115,028–122,573	Cyt
HSP70-1	ENSCSAVG00000009801	622/67.999/5.95	reftig_1:1998692:2000750	Mito
Hsp70-2	ENSCSAVG00000006429	657/72.054/5.11	reftig_140: 663,164–666,781	ER
Hsp70-3	ENSCSAVG00000004764	655/71.629/5.26	reftig_117: 237,857–240,895	Cyt
Hsp70-4	ENSCSAVG00000009296	647/71.617/5.61	reftig_11: 1,397,712–1,399,655	Nucl
Hsp70-5	ENSCSAVG00000008588	644/71.216/5.34	reftig_49: 1,564,826–1,572,778	Cyto_Nucl
Hsp70-6	ENSCSAVG00000010133	496/54.367/5.85	reftig_58: 2,768,858–2,774,321	Cyt
Hsp70-7	ENSCSAVG00000010360	775/87.079/6.21	reftig_49: 3,005,369–3,016,199	Cyt
Hsp70-8	ENSCSAVG00000009021	587/65.851/8.70	reftig_27: 631,835–635,495 -1	Nucl
Hsp100	ENSCSAVG00000003312	879/100.328	reftig_21: 370,847–391,331	Nucl
Hsp90-1	ENSCSAVG00000002810	721/82.703/4.82	reftig_11: 207,906–212,165	Cyt
Hsp90-2	ENSCSAVG00000010760	817/93.408/4.63	reftig_14: 1,223,243–1,229,985	ER
Hsp90-3	ENSCSAVG00000005261	714/81.136/5.64	reftig_76: 499,897–510,992	Mito


*AA, amino acid; MW, molecular weight; PI, isoelectric point; Cyt, cytoplasm; ER, endoplasmic reticulum; Mito, mitochondrion; Nucl, nucleus.*

Similarly, we found 31 putative Hsp and Hsf genes in the genome of *C. robusta* including three Hsp20, six Hsp40, ten Hsp60, seven Hsp70, three Hsp90, one Hsp100, and one Hsf (Supplementary Table S1). In addition to highly similar gene number, both the subcellular localization and the composition of gene family were similar to those of *C. savignyi* (Supplementary Table S1).

### Phylogenetic Relationship, Gene Structure, and Conserved Domain

**FIGURE 1:**
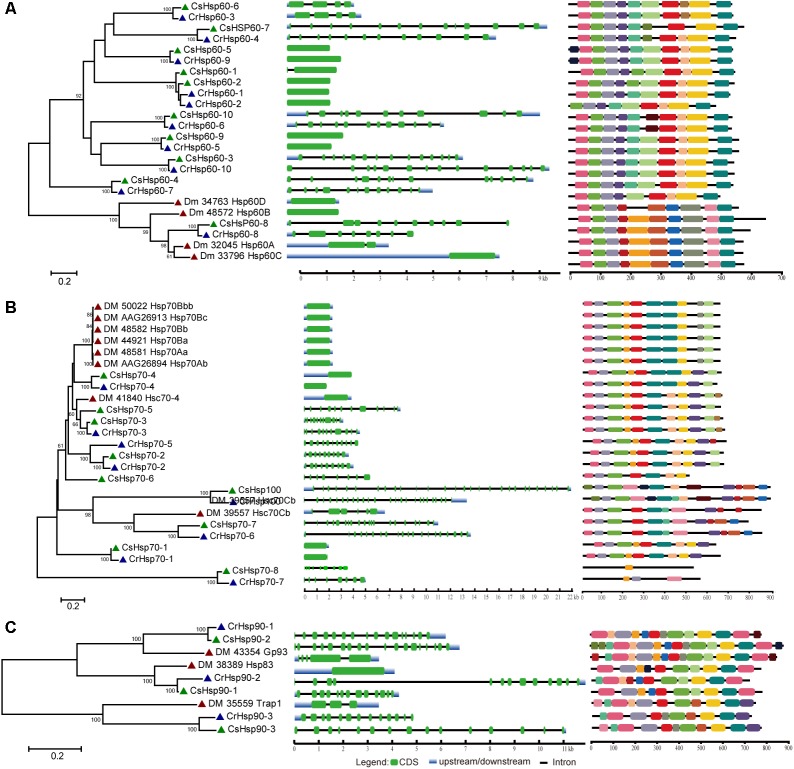
Phylogenetic relationship, gene structure and motif composition of Hsp60 **(A)**, Hsp70 **(B)**, and Hsp90 **(C)** in *C. robusta* (Cr: navy triangle), *C. savignyi* (Cs: green triangle), *D. melanogaster* (Dm: red triangle). Phylogenies were reconstructed using MEGA 6.0 by the maximum likelihood (ML) method with 1,000 bootstrap replicates (left panel). Exon/intron structure is shown in the mid panel, green boxes represent exons, blue boxes represent untranslated regions, and black lines represent introns. A schematic representation of conserved motifs (obtained using MEME) in Hsp60, Hsp70, and Hsp90 proteins is shown in the right panel. Different motifs are represented by boxes of different colors.

Regarding the relative importance and gene number of Hsp families, we performed the phylogenetic analysis of Hsp60, Hsp70/Hsp100, and Hsp90 families based on the full length peptide sequences of *C. savignyi*, *C. robusta*, *D. melanogaster*, *C. elegans*, *B. floridae*, *P. marinus*, *D. rerio*, and *X. laevis* to investigate their potential evolutionary relationships (Supplementary Figure S1). The *Ciona* Hsp were grouped with their corresponding othologs from other species in various subfamilies, indicating the evolutionary conservative but functional diversity. To further study the evolution of gene and protein structures, *D. melanogaster* was chosen to construct phylogenetic trees with *C. robusta* and *C. savignyi*. The results showed that Hsp60 family was divided into two major clusters corresponding to two subfamilies of Hsp60, CCT/TRiC, and Hsp60 homologous to bacterial GroEL (**Figure 1A**, left panel). We found four pairs of orthologous proteins (CsHsp60-7/CrHsp60-4, CsHsp60-4/CrHsp60-7, CsHsp60-6/CrHsp60-3, CsHsp60-8/CiHsp60-8) in *C. savignyi* and *C. robusta* (**Figure 1A**, left panel), among which the first three pairs had similar gene structure (**Figure 1A**, mid panel) and the last pair had the same cellular localization (**Table 1** and Supplementary Table S1). For the Hsp70 family, paralogous proteins (DmHsp70Bc, DmHsp70Bb, DmHsp70Bbb, DmHsp70Ba, DmHsp70Aa, and DmHsp70Ab) were grouped into a clade (**Figure 1B**, left panel), mainly as the Hsp70 family in *D. melanogaster* expanded by tandem duplications. Most orthologous proteins in *C. savignyi* and *C. robusta* (CsHsp70-1/CrHsp70-1, CsHsp70-2/CrHsp70-2, CsHsp70-3/CrHsp70-3, CsHsp70-4/CrHsp70-4, CsHsp70-7/CrHsp70-6, CsHsp-8/CrHsp70-7, CsHsp100/CrHsp100) shared the same or similar gene structures (e.g., exon/intron number; **Figure 1B**, mid panel), cellular localization and motif composition (**Figure 1B**, right panel), suggesting that orthologous Hsp70 genes should be more evolutionarily conserved than the paralogs in their genomes. For the Hsp90 family, each CsHsp90 member had orthologous gene in *C. robusta* and *D. melanogaster* (**Figure 1C**, left panel). *Ciona* Hsp90 had fewer introns than DmHsp90 (**Figure 1C**, mid panel), and each sister branch shared conserved motifs, especially for the last motif in the C-terminal of proteins (**Figure 1C**, right panel). The functional MEEVD motif features for cytosolic Hsp90 were presented in the C-terminal of CsHsp90-1, CrHsp90-2, and DmHsp83, which were predicted to be located in cytoplasm. In additional, C-terminal ER-retention HDEL motif was presented in ER-localized CsHsp90-2, CrHsp90-1, and DmGp93. CsHsp90-3 and its orthologs were localized in mitochondria although the typical transit peptide for importing into mitochondria was not found in them, and this cluster was designated to TRAP1 (tumor necrosis factor receptor-associated protein 1) subfamily of Hsp90.

### Heat-Shock *Cis*-Regulatory Elements Detection

**FIGURE 2:**
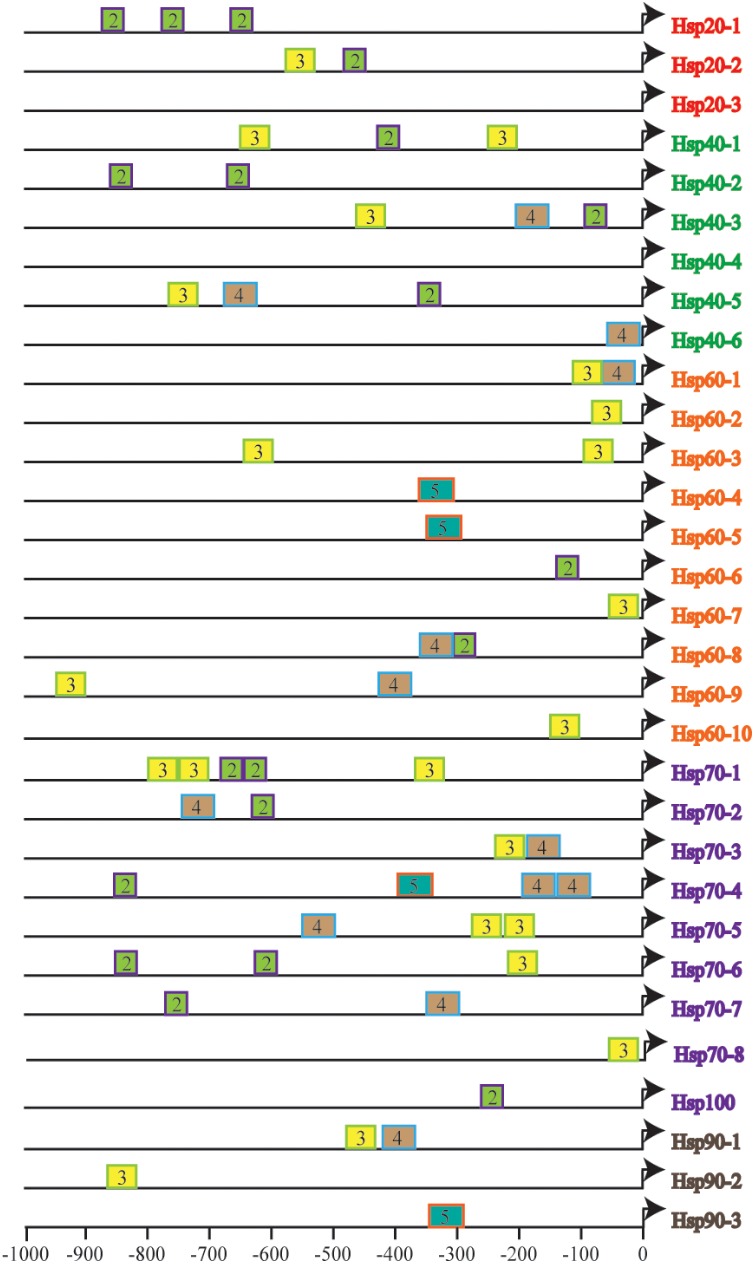
The number and distribution of identified HSEs in 31 CsHsps. Triangles represent transcription start site (TSS), and rectangles represent HSE. The number in the rectangles indicates length of HSE in 5 bp (nGAAn/nTTCn) units, and different colors of rectangles represent different length of HSE.

To further understand the fine tuning of heat shock response (HSR) through recognization of Hsf to HSE in the promoters of various Hsp genes, we estimated the size, number, context and their distance from the TSS of HSE in the identified CsHsp (**Figure 2**), all of which determined the strength of promoters. A total of 58 HSE (36 tail types and 22 head types, starting with “nTTCn” and “nGAAn” subunits, respectively) were identified across 29 CsHsp, with no HSE detected in CsHsp20-3 and CsHsp40-4, indicating that alternative regulatory mechanisms might underlie these two genes mediated by HSR. The size, number, and position of HSE varied among different CsHsp genes (Supplementary Table S2). The largest size of HSE contained five GAA/TTC elementary units in CsHsp60-4, CsHsp60-5, CsHsp70-3, and CsHsp90-3. The CsHsp70 family exhibited the highest number of HSE, and the HSE in CsHsp60 genes were closest to TSS (**Figure 2**). For CrHsp, a total of 90 HSE (29 tail types “nTTCN” and 61 head types “nGAAn”) were identified across 30 Hsp, with different HSE numbers in each Hsp gene (Supplementary Table S3 and Supplementary Figure S2). Larger size of HSE was detected in CrHsp40-1 (eight units), CrHsp60-9 (nine units), CrHsp70-4 (nine and seven units) than corresponding CsHsp (Supplementary Table S3 and Supplementary Figure S2). We also detected potential HSE within 1,000 bp downstream of TSS, which might also play important roles in Hsf–Hsp interactions (Supplementary Tables S2, S3).

### Expression Changes of CsHsp and CsHsf After Environmental Stresses

**FIGURE 3:**
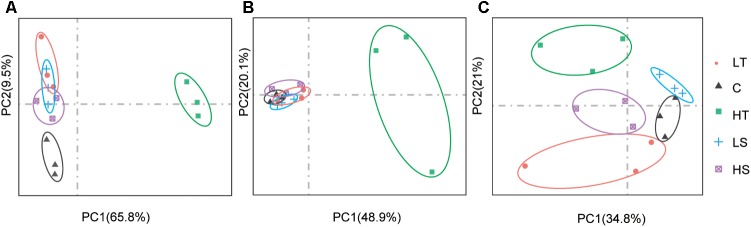
Principal component analysis (PCA) analysis of gene expression based on the fragments per kilo-base per million reads (FPKM) values of CsHsp and CsHsf genes at 1 h **(A)**, 24 h **(B)**, and 48 h **(C)** after different stress treatments. C, control group; LT, low temperature; HT, high temperature; LS, low salinity; HS, high salinity.

**FIGURE 4:**
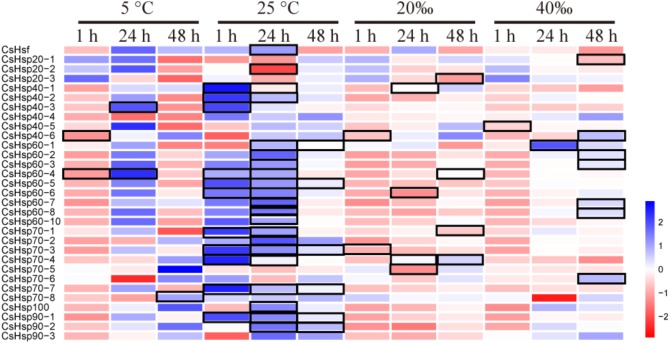
Expression profiles of CsHsps and CsHsf under low temperature (5°C), high temperature (25°C), low salinity (20aaa), and high salinity (40aaa) stresses for 1-, 24-, and 48-h, respectively. Color scale represents log_2_ fold change value of the expression in treatment group compared with control group, and black frame represents significant changes of gene expression (*P* < 0.05).

On average, four giga base raw data was generated for each sample in the RNA-Seq experiment (GenBank accession number: SRP152910). After trimming the raw data, approximately 80% of clean data was successfully mapped to the reference genome, and all predicted genes in genome were assembled using StringTie. The PCA analysis revealed clear gene expression differentiation between treatment and control groups at all three time points, explaining 75.3%, 69.0%, and 55.8% of the total variation at the expression level by two major axes (**Figure 3**). At both 1 and 48 h, all four experimental groups were clearly separated from their corresponding control groups (**Figures 3A,C**), and only high temperature treatment was separated from control group at 24 h (**Figure 3B**). To investigate the possible roles of Hsp and Hsf in *C. savignyi*’s stress response, gene expression profiles of 31 genes (except for the novel gene CsHsp60-9) were analyzed under temperature and salinity stresses. Overall, not all CsHsp could be induced by the four types of stresses. For example, CsHsp90-3 and CsHsp40-4 did not show significant changes at any time points under any type of stresses (Supplementary Table S4). The maximal range of genes (21/30) were induced significantly at 24-h after the high temperature stress, and the maximum amplitude of fold change occurred in CsHsp70-4 after 1-h of the high temperature treatment (up-regulation by 4734 folds; **Figure 4** and Supplementary Table S4). Some Hsp such as CsHsp90-1 and CsHsp90-2 responded to one specific stress, while other genes responded to multiple stresses. No common differentially expressed genes (DEGs) were involved in all four environmental stresses simultaneously (Supplementary Figure S3), while two DEGs (CsHsp60-4 and CsHsp40-3) responded to both low and high temperature stresses and another two DEGs (CsHsp40-5 and CsHsp40-6) were involved in both high and low salinity stresses (Supplementary Figure S3). Additionally, high temperature and low salinity stress responses overlapped at six DEGs (CsHsp70-1, CsHsp70-3, CsHsp70-4, CsHsp60-5, CsHsp60-6, and CsHsp40-1), and five DEGs (CsHsp60-1, CsHsp60-2, CsHsp60-3, CsHsp60-7, and CsHsp60-8) responded to high temperature and high salinity stresses. These common DEGs responsible for two or more stressors might play important roles in organisms’ adaptation to complex situations in the wild.

### Temporal Expression Patterns of Hsp and Hsf Under Stresses

**FIGURE 5:**
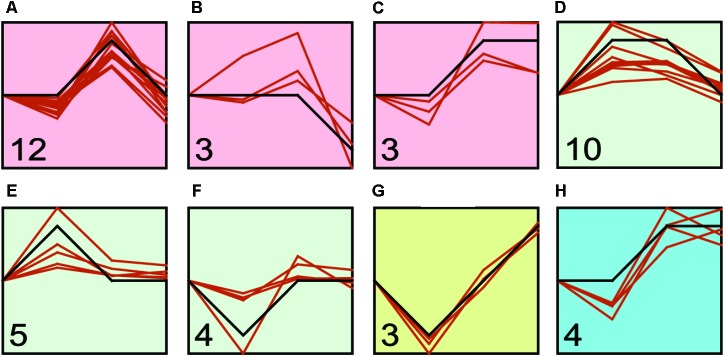
STEM profiles of the most significant clusters of CsHsp and CsHsf genes under low temperature **(A–C)**, high temperature **(D–F)**, low salinity **(G)**, and high salinity **(H)** stresses. The *X*-axis shows the time points (0-, 1-, 24-, and 48-h), and the *Y*-axis represents the log_2_ fold change of corresponding gene expression in treatment groups compared to control group. The number in the bottom left corner shows the number of genes clustered to this profile, while the orange curve shows individual gene expression profiles.

We found that transcriptional regulation was a highly dynamic process (**Figure 4** and Supplementary Table S4), and the expression of Hsp and Hsf changed with the challenge duration time. Therefore, we identified the major expression profiles across time duration under four treatments by STEM program. We identified three, three, one and one significant clusters in low temperature, high temperature, low salinity, and high salinity treatments, respectively (**Figure 5** and Supplementary Table S5). After the low temperature stress, the profile a containing 12 genes (six Hsp60, three Hsp40, two Hsp70, one Hsp90) showed an up-regulated expression peak at 24-h and recovery at 48-h (**Figure 5A** and Supplementary Table S5). After high temperature stress, the most represented profile d containing 10 genes (six Hsp60, two Hsp70, one Hsp90, and one Hsf) showed an up-regulation immediately after 1-h of heat stress, then such an up-regulation was maintained until 24-h and then recovered at 48-h (**Figure 5D** and Supplementary Table S5). A faster and more durable up-regulation pattern under high temperature stress indicated a more intensive challenge to *C. savignyi*. After low salinity stress, the gene expression changes varied and only three genes were down-regulated at 1-h and then gradually increased (**Figure 5G** and Supplementary Table S5). After high salinity stress, the significant profile including four genes showed a gradually increase in the first 24-h of stress treatment and then maintained up-regulated expression (**Figure 5H** and Supplementary Table S5).

### Potential Regulatory Relationship Between Hsp and Hsf Genes

**FIGURE 6:**
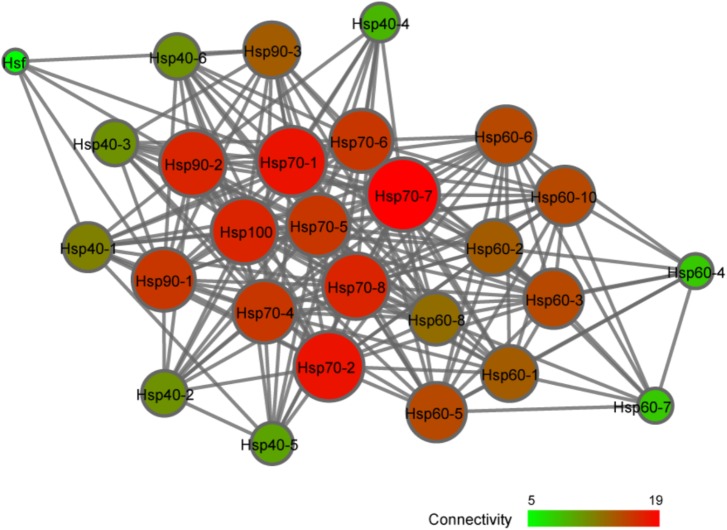
Interaction network analysis of Hsp and Hsf identified in *C. savignyi* by STRING program.

To further explore the possible regulatory relationship between CsHsp and CsHsf, an interaction network was constructed with STRING program. The single CsHsf gene, unexpectedly located at the edge of the network (**Figure 6**), interacted with only five Hsp (CsHsp90-1, CsHsp90-2, CsHsp90-3, CsHsp70-1, and CsHsp40-1). In addition to regulatory relationship between Hsf and Hsp, we detected many other interactions such as interactions between Hsp40 and Hsp70, between Hsp70 and Hsp90, and among subunits of Hsp60 chaperonins.

## Discussion

Biological invasions in marine and coastal ecosystems have become a global problem in the past three decades (Zhan et al., 2015). Successful invaders have abilities to rapidly adapt to changing environments during invasions, and such rapid environmental adaptation is considered as one of the most important determinants for invasion success (Zhan et al., 2015; Huang et al., 2017). At the molecular level, environmental stressors can potentially disturb normal folding of new proteins directly or indirectly, while pre-existing proteins may undergo denaturation and aggregation, leading to harms to cells and further survival of an organism (Szalay et al., 2007). Hsp can be functional as molecular chaperons that protect organisms against various stresses. Different from previous studies focusing on one or several Hsp families, we performed a genome-wide comprehensive survey, characterized all potential Hsp and Hsf across the whole genome, analyzed transcriptional expression variation of Hsp and Hsf in response to temperature and salinity stresses, and finally estimated potential regulatory relationship between Hsp and Hsf.

### The Number and Gene Structure of Hsp in the Genome of Genus *Ciona*

We identified only 31 and 30 Hsp after a comprehensive survey of *C. savignyi* and *C. robusta* genomes, respectively. When compared with other animals’ genomes, the composition of Hsp families was largely simplified but completely profiled. The teleost fishes such as zebra fish have more Hsp members in each gene family than ascidians mainly due to the whole-genome duplication events around 320–350 million years ago (Glasauer and Neuhauss, 2014; Borchel et al., 2017). Larger Hsp numbers for most Hsp gene families were also detected in *Mus musculus* and *Xenopus laevis* (Borchel et al., 2017) (Supplementary Figure S1). The Hsp70 families of many marine organisms living in highly fluctuating intertidal zones have significantly expanded mainly through the tandem duplication mechanism, for example, 88 copies in the Pacific oyster (*Crassostrea gigas*) (Zhang et al., 2012), 97 in the pearl oyster (*Pinctada fucata*) (Takeuchi et al., 2016) and 61 in the Yesso scallop (*Patinopecten yessoensis*) (Cheng et al., 2016). Despite relatively fewer Hsp genes identified in *Ciona* ascidians, all members of Hsp gene families exist in their genomes, suggesting that the evolutionary process of HSR system is conserved in composition but differentiated in duplicated numbers.

The lack of introns has been considered as a typical feature for most Hsp genes; however, multiple studies have found numerous exceptions (Evgen’ev et al., 2014). So far, available evidence showed mixed results of the roles of gene structure in Hsp gene expression. On one hand, because the splicing machinery is usually disturbed by various stresses, the Hsp transcript with no intron can rapidly move from nucleus to cytoplasm without splicing, leading to the preference for selection of intronless genes during the process of evolution (Evgen’ev et al., 2014). On the other hand, some studies showed that genes with introns allow for more mRNA accumulation than intronless genes (Le Hir et al., 2003). In our study, we did not find introns in 40% (4/10) of CsHsp60 and 25% (2/8) of CsHsp70 members, while other genes in Hsp60, Hsp70, and Hsp90 families had 1–27 introns (**Figure 1**). As multiple factors may affect gene expression, we could not conclude the relationship between the intron number and gene expression based on available evidence obtained here. However, the maximum amplitude of fold change after 1-h of high temperature treatment occurred in an intronless gene CsHsp70-4 (**Figure 1** and Supplementary Table S4).

### Different Response Pattern of CsHsp to Diverse Environmental Stresses

We observed stress-induced gene expression changes in most but not all CsHsp genes. In addition to CsHsp90-3 and CsHsp40-4 which stably expressed under all treatments, CsHsp70-5 and CsHsp70-6 were completely not induced by temperature stress but occasionally induced by salinity stress, indicating that these genes mainly function as constitutive proteins. Our results also showed that the induction of CsHsp expression was in duration- and stress-specific pattern. Most CsHsp including the most important stress response chaperon Hsp70 and Hsp90 members were not cold-inducible, except for CsHsp60-4, CsHsp40-3, and CsHsp40-6 (Supplementary Table S4). During the cold challenge on *D. melanogaster*, the mRNA expression of Hsp genes was also not regulated during cold stress but during the recovery phase because the protein denaturation process was unlikely to occur at low temperature (Colinet et al., 2010). Our RNA-seq analysis showed that the DEGs were enriched in the biological processes of negative regulation of apoptotic and programmed cell death (unpublished results), indicating that the main cellular process should be anti-apoptotic but not stress response process. Contrary to low temperature, most CsHsp were significantly induced by high temperature, and medium proportion of CsHsp was induced by salinity stress (**Figures 4, 5**). The Hsp expression changes after challenges showed a dynamic pattern (**Figures 4, 5**), among the eight significant profiles identified by STEM, four of which (**Figures 5A,D–F**) showed that stress-induced transcriptional changes returned to the control values after 24- or 48-h. Many studies found that the ability of gene expression recovery from stress-induced changes to a normal level was correlated with species’ stress resistance (Franssen et al., 2011), so the ability of CsHsp expression returning to the control level at 24- or 48-h might contribute to rapid adaptation during invasions of *C. savignyi*.

### Comparison of Stress Response Between *C. savignyi* and *C. robusta* Hsp

Two congeners belonging to the genus *Ciona* are both notorious invaders in marine ecosystems, and have been developed as model species for studying invasion success (Zhan et al., 2010, 2012, 2015). From their current global distributions, *C. robusta* is more widespread and can tolerate a wider range of water temperatures and salinities than *C. savignyi* (Serafini et al., 2011). In our study, after 1-h of high temperature stress, CsHsp70-4 showed highest induction among seven CsHsp70 members. Similarly, its counterpart in *C. robusta* CrHsp70-4 (Ci-HSPA1/6/7-like) also showed highest induction among all Hsp70 genes after 3-h under high temperature stress (28°C) (Fujikawa et al., 2010). Although studies showed that Hsp70 genes could be transcriptionally induced by heat stress in two ascidians (Fujikawa et al., 2010; Huang et al., 2016), a direct comparison between these two species in different studies seems inappropriate owing to the treatment difference. It was also reported that many Hsp could be induced by heat stress at the proteomic level in both ascidians; however, *C. robusta* maintained higher constitutive levels of Hsp and could respond faster to thermal stress than *C. savignyi*, which might partially explain the larger geographical distribution of *C. robusta* globally (Serafini et al., 2011). The different Hsp-based stress tolerance based on Hsp between lizard species resulted from not only different gene copy number but also the regulatory machinery of Hsf (Zatsepina et al., 2000). Regarding the fact that we identified exactly the same gene number of each gene family in *C. savignyi* and *C. robusta*, we argue that the process of binding Hsf to HSE potentially contributes to the difference of stress response in these two species. Multiple factors, including more HSE, larger subunit size, closer distance to TSS in *C. robusta* Hsp (**Figure 2** and Supplementary Figure S2), as well as different preferences for G-type or T-type HSE (Supplementary Tables S2, S3), might affect the power of Hsp promoters, leading to different tolerance levels of environmental stresses. More deep investigations using electrophoretic mobility shift assay (EMSA) or luciferase assay should be performed to experimentally verify functional activities of HSE identified in this study.

### The Regulatory Relationship Between Hsf and Hsp

Multiple Hsf genes (Hsf1-4, and HsfY) have been detected in vertebrates, while in genus *Ciona* only one Hsf gene was observed with one transcript. Despite only one Hsf in *D. melanogaster*, four differently spliced transcripts were detected (Zimarino et al., 1990). There are two ways for Hsf to respond to stresses – (1) protein activation and (2) transcription. Under normal conditions, the Hsf protein is present in the form of inactive monomers. When organisms were exposed to environmental challenges, Hsf is trimerized and subsequently translocated to nucleus for binding to HSE in Hsp promoters as a transcription factor (Vihervaara and Sistonen, 2014). In this scenario, Hsf transcriptional expression cannot be affected by stresses and maintain stably. However, Hsf can also be activated at the transcriptional level after stresses (Zhang et al., 2015), and then stress-induced up-regulation of Hsf mRNA was *de novo* translated to functional proteins to interact with Hsp genes, similarly to the up-regulation of CsHsf after 24-h of high temperature exposure in this study. Based on our results, we argue that these two mechanisms are not completely independent and both may be involved in regulating Hsp expressions. We observed few Hsf–Hsp interactions and weak correlations between Hsf and Hsp expression (**Figure 6**), suggesting that protein activation was possibly the main strategy adopted by Hsf to regulate Hsp expression in this study. In addition to Hsf, many other factors can affect Hsp-related stress response, such as modifications of histones and remodeling of chromatins (Guertin and Lis, 2010), which indirectly determined the Hsf binding activity. Additionally, many other proteins can participate in Hsp transcription, epigenetic modification or translation (e.g., eEF1A1) (Evgen’ev et al., 2014; Pu and Zhan, 2017). Collectively, stress response process involved in fine tuning of Hsp is a complex multi-level process, forming a rapid and flexible mechanism for organisms’ adaptation to changing environments.

## Conclusion

This study successfully identified and characterized a full set of Hsp and Hsf gene families in a marine invasive species, *C. savignyi*. A total of 32 genes including three Hsp20, six Hsp40, 10 Hsp60, eight Hsp70, three Hsp90, one Hsp100, and one Hsf were identified across the whole genome. Our results showed that most, but not all, Hsp could respond to one or more temperature or salinity-related environmental stresses, and the induction of CsHsp expression are in duration- and stress-specific pattern. The weak correlation between Hsf and Hsp expression suggests that protein activation should be the main strategy adopted by Hsf to regulate Hsp expression. Our results here provide clues for deeply understanding the binding activity of HSE and complex interactions between Hsf and Hsp during the biological response to multiple environmental challenges. In addition, the results here improve our knowledge on functions of Hsp and Hsf during molecular response to harsh environments, thus facilitating our understanding of molecular mechanisms of invasion success in aquatic ecosystems using *C. savignyi* as a model.

## Data Accessibility

The RNA-Seq raw sequence data was deposited in the National Center for Biotechnology Information (NCBI) Sequence Read Archive (SRA) database under the accession numbers SRP152910.

## Author Contributions

XH and AZ conceived and designed this study, and drafted the manuscript. SL and YG assisted with the experimental treatments. XH analyzed the data. All authors reviewed and commented on the manuscript.

## Conflict of Interest Statement

The authors declare that the research was conducted in the absence of any commercial or financial relationships that could be construed as a potential conflict of interest.

## Footnotes

1 http://www.uniprot.org/

2 http://pfam.xfam.org/

3 http://smart.embl-heidelberg.de/

4 http://web.expasy.org/protparam/

5 https://wolfpsort.hgc.jp/

6 http://gsds.cbi.pku.edu.cn/

7 http://meme-suite.org/tools/meme

8 http://www.genomatix.de/

9 http://www.string-db.org
