# Supplementary material for: Genome-Wide Identification, Characterization and Expression Analyses of Heat Shock Protein-Related Genes in a Highly Invasive Ascidian Ciona savignyi
Source: Front Physiol. 2018 Jul 31;9:1043. doi: 10.3389/fphys.2018.01043 (PMC6079275; doi:10.3389/fphys.2018.01043)
Supplement: Supplementary file 1 [file Data_Sheet_1.PDF]

# *Supplementary Material*

# Genome-Wide Identification, Characterization and Expression Analyses of Heat Shock Protein-Related Genes in A Highly Invasive Ascidian *Ciona savignyi*

**Xuena Huang[1,2], Shiguo Li[1,2], Yangchun Gao[1,2] and Aibin Zhan[1,2*]**

***Correspondence:** Prof. Aibin Zhan. E-mail: zhanaibin@hotmail.com; azhan@rcees.ac.cn.

**Supplementary material 2:**

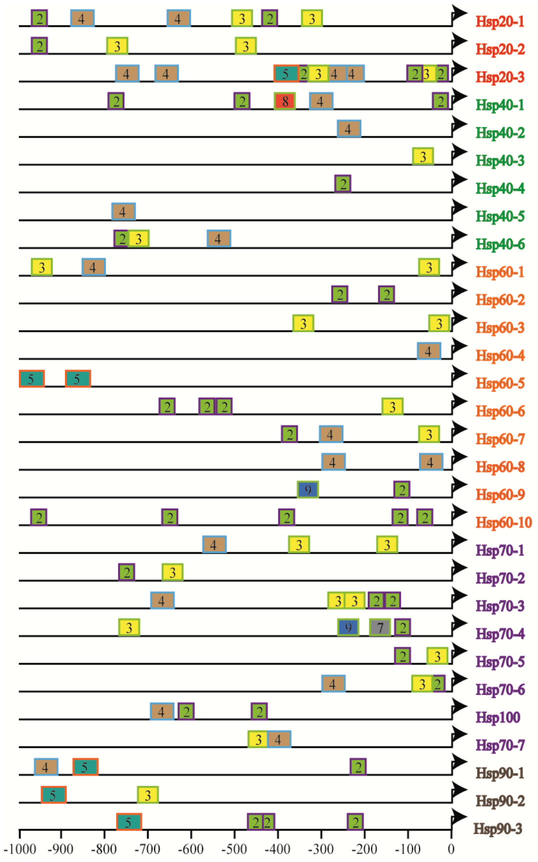


**Figure S1.** The number and distribution of identified heat shock elements (HSE) in 30 heat shock proteins (Hsp) in *Ciona robusta*. Triangles and rectangles represent transcription start site (TSS) and HSE, respectively. The number in rectangles indicates the length of HSE in 5 bp (nGAAn/nTTCn) units, and the different length of HSE is color-coded.

A

B

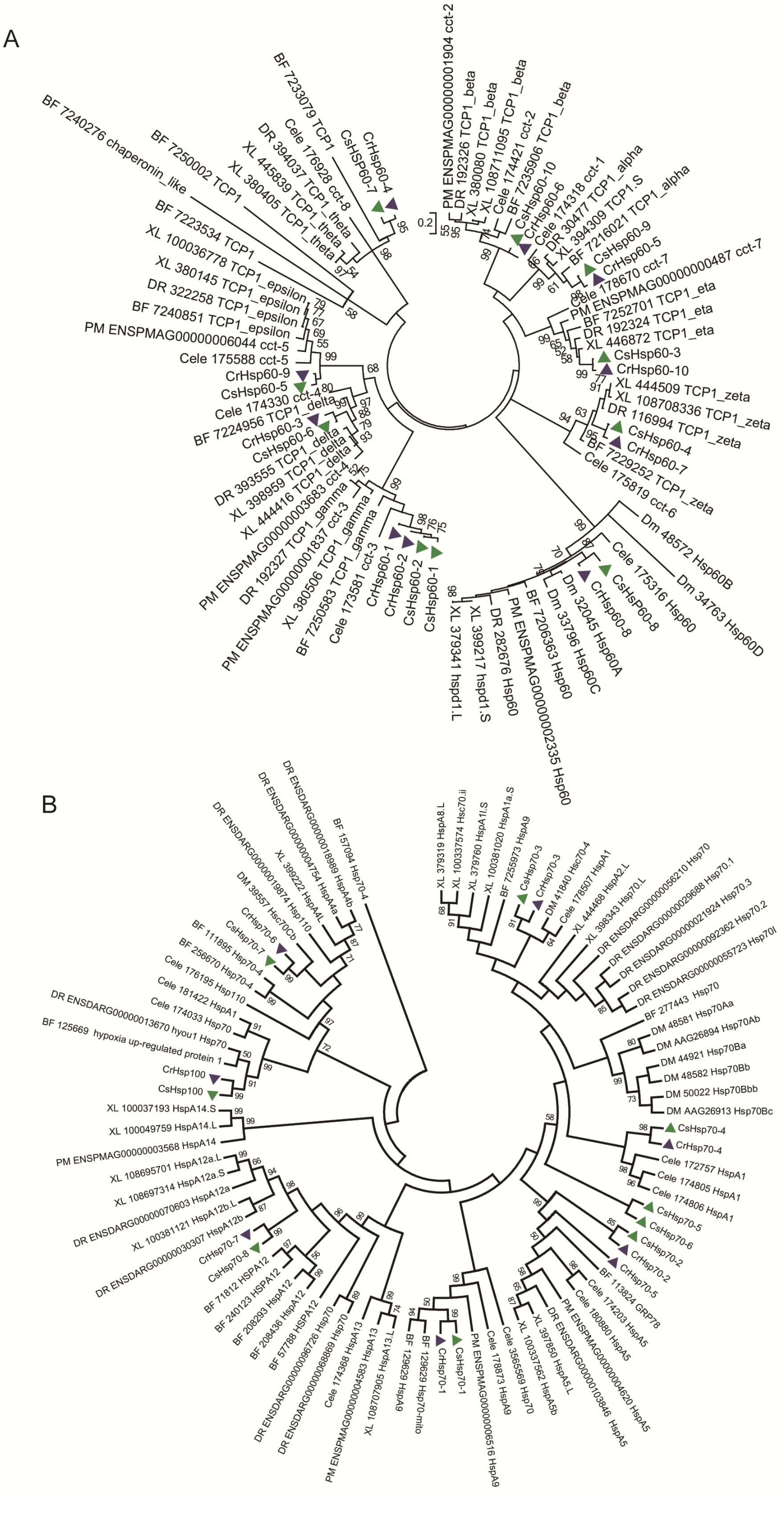

A
0.2
PM ENSPMAG00000001904 cct-2
DR 192326 TCP1_beta
XL 380080 TCP1_beta
XL 108711095 TCP1_beta
Cele 174421 cct-2
BF 7235906 TCP1_beta
CsHsp60-10
CrHsp60-6
Cele 174318 cct-1
DR 30477 TCP1_alpha
XL 394309 TCP1.S
BF 7216021 TCP1_alpha
CsHsp60-9
CrHsp60-5
Cele 178670 cct-7
PM ENSPMAG00000000487 cct-7
BF 7252701 TCP1_eta
DR 192324 TCP1_eta
XL 446872 TCP1_eta
CsHsp60-3
CrHsp60-10
XL 444509 TCP1_zeta
XL 108708336 TCP1_zeta
DR 116994 TCP1_zeta
CsHsp60-4
CrHsp60-7
BF 7229252 TCP1_zeta
Cele 175819 cct-6
Dm 48572 Hsp60B
Dm 34763 Hsp60D
Cele 175316 Hsp60
CsHsp60-8
CrHsp60-8
Dm 32045 Hsp60A
Dm 33796 Hsp60C
BF 7206363 Hsp60
PM ENSPMAG00000002335 Hsp60
DR 282676 Hsp60
XL 399217 hspd1.S
XL 379341 hspd1.L
CsHsp60-1
CsHsp60-2
CrHsp60-2
CrHsp60-1
Cele 173581 cct-3
BF 7250583 TCP1_gamma
XL 380506 TCP1_gamma
PM ENSPMAG00000001837 cct-3
DR 192327 TCP1_gamma
PM ENSPMAG00000003683 cct-4
XL 444416 TCP1_delta
XL 398959 TCP1_delta
DR 393555 TCP1_delta
CsHsp60-6
CrHsp60-3
BF 7224956 TCP1_delta
Cele 174330 cct-4
CsHsp60-5
CrHsp60-9
Cele 175588 cct-5
PM ENSPMAG00000006044 cct-5
BF 7240851 TCP1_epsilon
DR 322258 TCP1_epsilon
XL 380145 TCP1_epsilon
XL 100036778 TCP1_epsilon
BF 7223534 TCP1
BF 7240276 chaperonin_like
BF 7250002 TCP1
XL 380405 TCP1_theta
XL 445839 TCP1_theta
DR 394037 TCP1_theta
Cele 176928 cct-8
BF 7233079 TCP1
CsHSP60-7
CrHsp60-4
B
BF 157094 Hsp70-4
DR ENSDARG00000018989 HspA4b
DR ENSDARG00000004754 HspA4a
XL 399222 HspA4L
DR ENSDARG00000019874 Hsp110
DM 39557 Hsc70Cb
CrHsp70-6
CsHsp70-7
BF 111895 Hsp70-4
BF 256670 Hsp70-4
Cele 176195 Hsp110
Cele 181422 HspA1
DR ENSDARG00000013670 hyou1 Hsp70
Cele 174033 Hsp70
BF 125669 hypoxia up-regulated protein 1
CrHsp100
CsHsp100
XL 100037193 HspA14.S
XL 100049759 HspA14.L
PM ENSPMAG00000003568 HspA14
XL 108695701 HspA12a.L
XL 108697314 HspA12a.S
XL 108697314 HspA12a
DR ENSDARG00000070603 HspA12b.L
XL 100381121 HspA12b
DR ENSDARG00000030307 HspA12
CrHsp70-7
CsHsp70-8
BF 71812 HSPA12
BF 240123 HspA12
BF 209293 HspA12
BF 208436 HspA12
BF 57788 HspA12
DR ENSDARG00000096726 Hsp70
DR ENSDARG00000068869 HspA13
Cele 174368 HspA13
PM ENSPMAG00000004583 HspA13
XL 108707905 HspA13.L
BF 129629 HspA9
BF 129629 Hsp70-mito
CrHsp70-1
CsHsp70-1
PM ENSPMAG00000006516 HspA9
Cele 178873 HspA9
Cele 3565569 Hsp70
XL 100337582 HspA5.L
XL 397850 HspA5b
DR ENSDARG00000103846 HspA5
PM ENSPMAG00000004620 HspA5
Cele 180880 HspA5
Cele 174203 HspA5
BF 113824 GRP78
CrHsp70-5
CrHsp70-2
CsHsp70-2
CsHsp70-6
CsHsp70-5
Cele 174806 HspA1
Cele 174805 HspA1
Cele 172757 HspA1
CrHsp70-4
CsHsp70-4
DM AAG26913 Hsp70Bc
DM 50022 Hsp70Bbb
DM 48582 Hsp70Bb
DM 44921 Hsp70Ba
DM AAG26894 Hsp70Ab
DM 48581 Hsp70Aa
BF 277443 Hsp70
DR ENSDARG00000055723 Hsp70l
DR ENSDARG00000092362 Hsp70.2
DR ENSDARG00000021924 Hsp70.3
DR ENSDARG00000029688 Hsp70.1
DR ENSDARG00000056210 Hsp70
XL 398343 Hsp70.L
XL 444468 HspA2.L
Cele 178507 HspA1
DM 41840 Hsc70-4
CrHsp70-3
CsHsp70-3
BF 7255973 HspA9
XL 100381020 HspA1a.S
XL 379760 HspA1l.S
XL 100337574 Hsc70.ii
XL 379319 HspA8.L

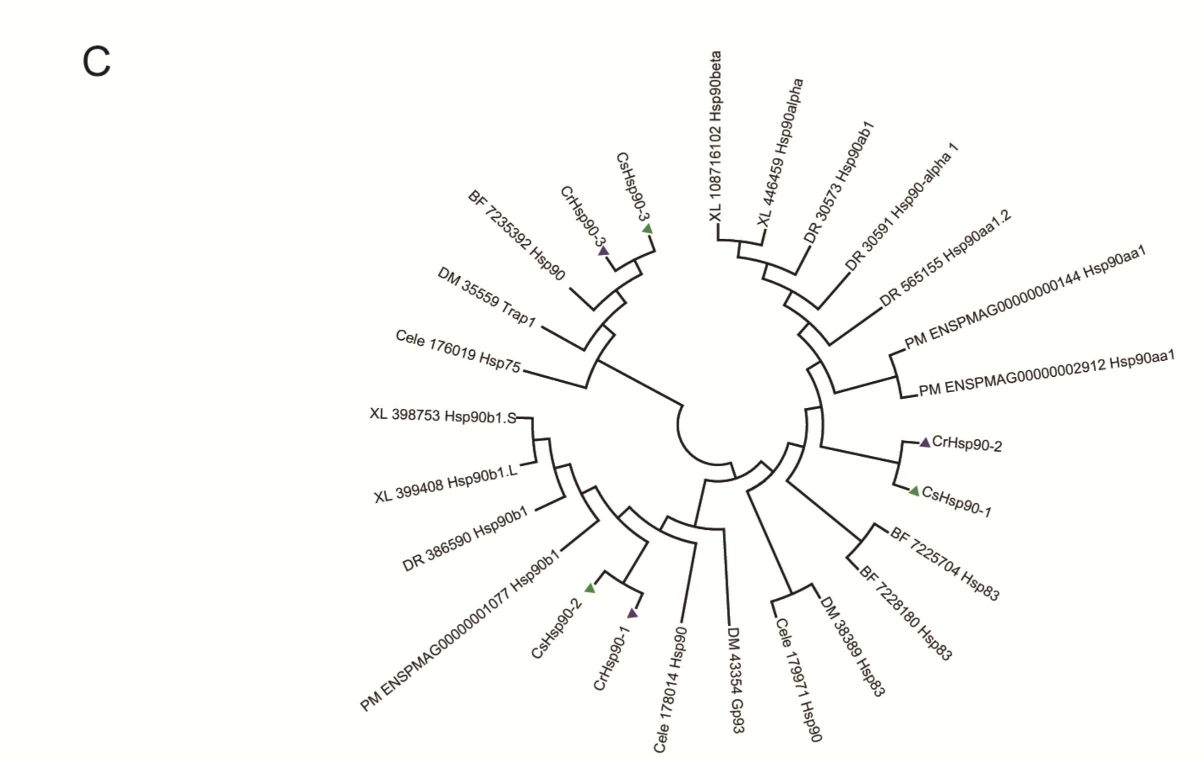


**Figure S2.** Molecular phylogenetic relationship of Hsp60 (figure A), Hsp70 (figure B) and Hsp90 (figure c) families in 8 species. The trees were reconstructed using the full length protein sequences by Maximum Likelihood method based on JTT model, with 1000 bootstrap replicates (bootstrap support value less than 50% are not shown). The abbreviation of the species in the figure are as follows: Cele (*Caenorhabditis elegans*), Cs (*Ciona savignyi*), Cr (*Ciona robusta*), DM (*Drosophila melanogaster*), BF (*Branchiostoma floridae*), PM (*Petromyzon marinus*), DR (*Danio rerio*), XL (*Xenopus laevis*). Species names are followed by gene ID and gene names. *C. savignyi* and *C. robusta* genes are marked by green and blue triangles, respectively.

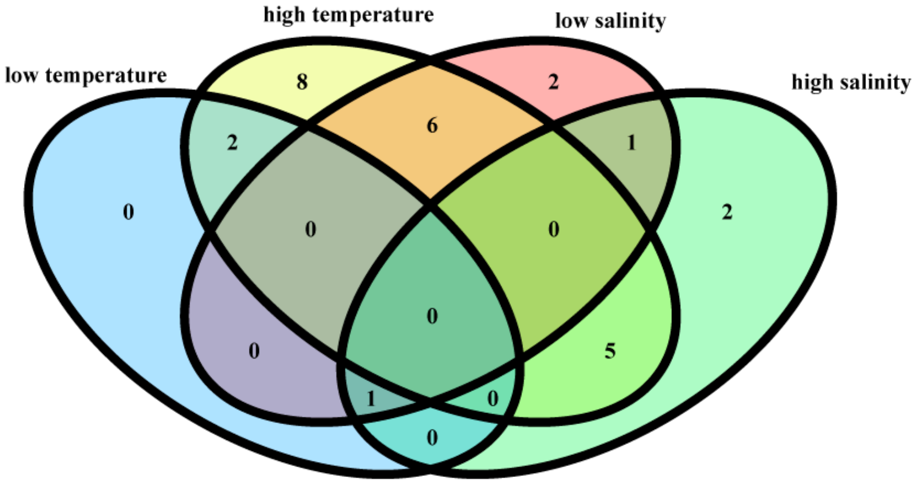


**Figure S3.** The number of differentially expressed genes after temperature and salinity stresses.
